# Gut Microbiota: Its Potential Roles in Pancreatic Cancer

**DOI:** 10.3389/fcimb.2020.572492

**Published:** 2020-10-07

**Authors:** Quanxiao Li, Meng Jin, Yahui Liu, Limin Jin

**Affiliations:** ^1^Department of Hepatobiliary and Pancreatic Surgery, The First Hospital of Jilin University, Changchun, China; ^2^Department of Radiation Oncology, The First Affiliated Hospital of Sun Yat-sen University, Guangzhou, China; ^3^Department of Anesthesia, The First Hospital of Jilin University, Changchun, China

**Keywords:** gut microbiota, pancreatic cancer, inflammation, immune response, immunotherapy

## Abstract

Pancreatic cancer is considered a lethal disease with a low survival rate due to its late-stage diagnosis, few opportunities for resection and lack of effective therapeutic strategies. Multiple, highly complex effects of gut microbiota on pancreatic cancer have been recognized as potential strategies for targeting tumorigenesis, development and treatment in recent decades; some of the treatments include antibiotics, probiotics, and fecal microbiota transplantation. Several bacterial species are associated with carcinogenesis of the pancreas, while some bacterial metabolites contribute to tumor-associated low-grade inflammation and immune responses via several proinflammatory factors and signaling pathways. Given the limited evidence on the interplay between gut microbiota and pancreatic cancer, risk factors associated with pancreatic cancer, such as diabetes, chronic pancreatitis and obesity, should also be taken into consideration. In terms of treatment of pancreatic cancer, gut microbiota has exhibited multiple effects on both traditional chemotherapy and the recently successful immunotherapy. Therefore, in this review, we summarize the latest developments and advancements in gut microbiota in relation to pancreatic cancer to elucidate its potential value.

## Highlights

- Alterations in gut microbiota in the human intestinal tract influence the initiation and development of pancreatic cancer.
- Chronic inflammation and the immune response generated by microbial metabolites modulate biological activities of pancreatic cancer.
- Gut microbiota also impacts pancreatic cancer *via* diabetes, obesity, and chronic inflammation.
- Treatments for pancreatic cancer could also affect gut microbiota.

## Introduction

Pancreatic cancer (PC) is commonly regarded as one of the most fatal diseases owing to its high malignancy and poor prognosis. According to the latest data, the 5-year overall survival rate of PC has increased slightly, but remains <10% (American Cancer Society, 2020). Rare representative symptoms and diagnosis of PC are considered key reasons why the mortality is close to its incidence (Kamisawa et al., 2016). Surgical resection is the only potential cure for patients with localized PC, but no more than 20% of whom are eligible for initial resection. With these shortcomings, other substitutive therapies are highlighted for early metastasis or recurrence. However, treatment resistance lowers the therapeutic effectiveness and limits clinical utility. Taken together, an earlier diagnosis and implementation of efficient strategies appear to be increasingly beneficial.

As the most neglected system in the human body, gut microbiota (GM) has received increasing attention in recent years. GM comprises up to trillions of microbes within the human intestinal tract (Costello et al., 2009; Sekirov et al., 2010). Data on the human fecal microbial metagenome showed that an aggregate of 9.9 million microbial genes across these fecal microbiomes have been identified (Li et al., 2014). Despite several studies on the genetic susceptibility of dysbiosis on mice transplanted with disease-associated GM, the question remains open as to whether GM dysbiosis is a consequence or a cause of human diseases (Lynch and Pedersen, 2016). Influenced by age, dietary habits, antibiotic intake and other environmental factors, GM has been found to have a crucial impact on obesity, diabetes mellitus and even carcinogenesis. The Human Microbiome Project Part 2 has explored the mechanisms of GM in inflammatory bowel disease (Lloyd-Price et al., 2019), type 2 diabetes mellitus (T2DM) and prediabetes (Zhou et al., 2019). Nevertheless, until now, what is emerging and has been discovered regarding the effect of GM on PC is just a tip of the iceberg. This review will elucidate the latent causality and underlying relevance between PC and GM.

## Influence of GM on PC

### Alterations of Microbial Metabolites

Despite the colonization of the human gastrointestinal tract, only 10 are determined as carcinogenesis by International Agency for Cancer Research (de Martel et al., 2012; IARC Working Group on the Evaluation of Carcinogenic Risks to Humans, 2012), which indicates complex relations existing among GM, environmental triggering factors and cancer initiation (Karpinski, 2019).

There is flourishing evidence demonstrating changes in physical characteristics resulting from GM overgrowth (Shin et al., 2019). GM disorders reduce the thickness of mucus layer, which is always followed by a decreased antimicrobial defense and fewer gut peptides from L-cells and limited short chain fatty acids (SCFAs) (Cani, 2018). As a consequence, transformations of relative microbial metabolites lead to subsequent chain reactions. The reduced gut peptides, such as glucagon-like peptide-1 and peptide YY, play regulatory roles in food intake and glucose metabolism. Moreover, PPAR-γ inactivation resulting from lack of SCFAs requires higher oxygen available for the microbiota at the proximal mucosa and promotes Enterobacteriaceae proliferation (Philipson et al., 2013). Despite with low concentrations, bacterial metabolites, such as lipopolysaccharides (LPS), flagellin and SCFAs, can reach the circulation and distant organs *via* paracellular diffusion or co-transport with chylomicrons. To be specific, LPS, an important cytoderm component of gram-negative bacteria, can interact with several Toll-like receptor (TLR) signaling pathways with a distinct structural composition from other bacterial taxa (Backhed et al., 2003). Furthermore, lipoteichoic acid (LTA), a surface component of gram-positive bacteria and a key virulence factor, has been found to trigger the over-secretion of proinflammatory factors by binding to CD14 or TLR2 (Hermann et al., 2002). This activity may result in the ultimate development of PC, as illustrated by its involvement in chronic pancreatitis progression on mice with infection of *Enterococcus faecalis* (Maekawa et al., 2018). Another microbial metabolite generated by 7α-dehydroxylating bacteria and secondary bile acids, deoxycholic acid (DCA), was shown to enhance the induction of PC (Cao et al., 2017; Wang et al., 2019). DCA also accelerates the senescence-associated secretory phenotype (Saretzki, 2010) and the progression of intestinal cancer through increasing DNA damage and genome instability (Louis et al., 2014). Moreover, DCA-induced activation of the EGFR ligand, amphiregulin was identified as an oncogenic factor in both colorectal and PC by EGFR, mitogen activated protein kinase (MAPK) and STAT3 signaling pathways (Nagathihalli et al., 2014). Acetate, propionate and butyrate are typical SCFAs. For instance, butyrate can interfere histone modifications and transcriptional regulation (Cani and Jordan, 2018). Additionally, a decrease in propionate lowers the abundance of mucosal-associated invariant T cells and regulatory T cells guarding the intestinal lamina propria (Cani, 2018) (Table 1).

**Table 1:** The effects of microbes, bacterial components and their metabolites in pancreatic cancer.

**Item**	**Content**	**Description**	**Mechanism**	**References**
Bacterial components or metabolites	Lipopolysaccharides	An important cytoderm component of gram-negative bacteria	Interact with several Toll-like receptor signaling pathways with a distinct structural composition from other bacterial taxa	Backhed et al., 2003
	Lipoteichoic acid	A key virulence factor on the gram-positive bacteria surface	Trigger the over-secretion of proinflammatory factors by binding to CD14 or TLR2	Hermann et al., 2002
	Deoxycholic acid	A kind of secondary bile acid generated by 7α-dehydroxylating bacteria from a high dietary fat intake	Accelerate the senescence-associated secretory phenotype and the progression of intestinal cancer via promoting DNA damage and genome instability and activation of the EGFR ligands amphiregulin	Saretzki, 2010; Louis et al., 2014; Nagathihalli et al., 2014
	Short chain fatty acids	Fermented dietary fiber in intestinal tract, including acetate, propionate, and butyrate	Stimulate the secretion of gut peptides involved in food intake or glucose metabolism	Vatanen et al., 2018
			Butyrate inhibits histone deacetylases via interfering histone modifications and transcriptional regulation	Cani and Jordan, 2018
			Propionate decreases the abundance of mucosal-associated invariant T cells and Treg guarding inside the intestinal lamina propria	Cani, 2018
	Cytolethal distending toxin	Produced by proteobacteria	Participate in genetic alterations and induce formation of endoreduplication or hyperploidy even in the absence of cell division	Nougayrede et al., 2005
	Cyclomodulins	A growing family of bacterial molecules	Cause carcinogenesis through the active interference with host cell cycle	Nougayrede et al., 2005
	Cytotoxic necrotizing factor	A prevalent virulence determinant exclusively confined to *E. coli* phylogroup B2	Lead to the uncontrolled proliferation of cancer cells	Nougayrede et al., 2005
Certain typical bacteria	*Enterobacteriaceae*	Natural inhabitants in the human intestine implicated in intestinal and extraintestinal illnesses	Promote proliferation by PPAR-γ that requires higher oxygen available for the microbiota at the proximal mucosa	Philipson et al., 2013
	*Enterococcus faecalis*		Aggravate chronic pancreatitis and damage pancreas tissue by the stimulation inflammatory cytokines	Maekawa et al., 2018
	*H. pylori*	An initiating factor of kinds of gastrointestinal cancer	Increase the risk of pancreatic cancer relating to gastric ulcer via the greater endogenous nitrosation and the inflammatory response to ulcer development and healing process	Bao et al., 2010
	*Porphyromonas gingivalis*	The most prevalent oral microorganism for periodontal disease	Its associated serum level of IgG is positively related to the risk of PC	Ahn et al., 2012
	*Fusobacterium spp*	A group of anaerobic bacterium colonizing oral cavity	Remain malignant potential in the development of pancreatic cancer with the 8.8% presence	Mitsuhashi et al., 2015
	*Bifidobacteria*	The dominant bacterial populations in the gastrointestinal tract interacted in maturation of the immune system and use of dietary components	Induct tumor-specific T cell and increase CD8 (+) T cell numbers in the tumor microenvironment combined with anti-PD-L1 immunomodulator	Sivan et al., 2015
	*Bacteroides*	One of the most abundant bacterial phyla in the human gut breaking down host dietary and mucosal polysaccharides	Assist *Escherichia coli* to improve the tumorigenic effectiveness via triggering damage of double-stranded DNA	Cougnoux et al., 2014

Several bacterial toxic productions have been identified as possible carcinogenetic molecules. Colibactin and *Bacteroides* fragilis toxin assist *Escherichia coli* to improve the tumorigenic effectiveness *via* triggering damage of double-stranded DNA (Cougnoux et al., 2014). Significantly, cyclomodulins, a growing family of bacterial molecules, play putative roles in tumorigenesis through the active interference with host cell cycles. Cytotoxic necrotizing factor from *E. coli* and CagA lead to the uncontrolled proliferation of cancer cells, while cytolethal distending toxin and cycle inhibiting factor act as predisposing factors of tumorigenesis by participating in genetic alterations and inducing formation of endoreduplication or hyperploidy even in the absence of cell division (Nougayrede et al., 2005).

### Alterations of Specific Microbial Species in Human Intestine

Growing evidence demonstrates that GM affects the outcomes of PC (Aykut et al., 2019; Wei et al., 2019). Compared to the short-term survivors, long-term PDAC survivors possessed higher tumor microbial diversity and more composition, which is presented as a predict on PDAC survival (Riquelme et al., 2019). *H. pylori* may be an indispensable issue for the initiation and progression of PC. The increasing risk of PC relating to gastric ulcer found in a prospective cohort study involving 51,529 male subjects might have resulted from the greater endogenous nitrosation and the inflammatory response due to *H. pylori* infection (Bao et al., 2010). Additionally, given the overlap of oral microbiomes in the intestine, bacterial translocation from oral cavity or intestinal tract is also closely associated with PC (Whitmore and Lamont, 2014). For instance, *Fusobacterium spp*, a group of anaerobic bacterium colonizing the oral cavity, has been demonstrated a potentially prognostic biomarker of PC with the 8.8% presence (Mitsuhashi et al., 2015). Large prospective studies have reported a higher risk of PC in patients with higher concentrations of *Porphyromonas gingivalis* (Fan et al., 2018) and a reduced risk for increased levels of specific antibodies against commensal oral bacteria (Michaud et al., 2013) (Table 1).

Currently, compared with normal tissue of pancreas, a 1000-fold increase in intrapancreatic bactibilia was observed in pancreatic ductal adenocarcinoma (PDAC) specimens (Dickson, 2018). This finding is widely divergent from the previous assumption of a sterile pancreas (Wei et al., 2019; Nejman et al., 2020). Data from a constituent analysis of microbes harboring in pancreatic cyst fluid characterized this specific microbiota ecosystem and demonstrated microbiota harboring in the pancreas; the results showed that some taxa with possible deleterious effects within this niche may lead to the pancreatic neoplastic process (Li et al., 2017). Notably, it is the bacterial composition, rather the bacterial abundance, within the pancreas that correlates with pancreatic carcinogenesis. Therefore, the mechanism by which microbes flow toward and penetrate the pancreas is a key issue. The identification of *Enterococcus* and *Enterobacter* species primarily found in bile indicate a possible pathway where GM is transported toward the pancreatic tissue (Maekawa et al., 2018). Additionally, Pushallkar et al. demonstrated the accessibility of gut bacteria to pancreas, suggesting their direct effect on the microenvironment around the pancreas (Pushalkar et al., 2018).

### Microbiota-Driven Chronic Inflammation

In the past several decades, PC has been regarded as an inflammation-driven disease. However, explorations of details underlying chronic inflammation and PC are still ongoing. The tumorigenesis-promoting effects of the transportation of bioactive molecule to the tumor microenvironment, such as growth, survival and proangiogenic factors, were confirmed, which was unanticipated and counter to the previous understanding of tumor-associated inflammation (Grivennikov et al., 2010). Hence, inflammation is proposed as an emerging hallmark of cancer (Hanahan and Weinberg, 2011). Moreover, compelling investigations shed light on the important contributions of mutant Kras influenced by inflammation and changes in GM to the key activities related to oncogenesis. Despite its common mutation in ~90% of PC cases, the activation of Kras still requires hyperstimulation from LPS-driven inflammation (Daniluk et al., 2012). The activated Kras subsequently accelerates carcinogenesis by activating nuclear factor kappa B (NF-κB) pathway components, including inhibitor of NF-κB kinase 2 and cyclooxygenase 2 (Huang et al., 2014). Another study supplied evidence that chronic inflammation in pancreatic tissue triggers the oncogenic mutation of Kras in insulin-positive endocrine cells and induces dedifferentiation of functional epithelial cells, which results in PDAC (Gidekel Friedlander et al., 2009). Additionally, periodontal inflammations, commonly including gingivitis and periodontitis, are considered to cause systemic inflammation (Chang et al., 2016). *Porphyromonas gingivalis* is the most prevalent oral microorganism in periodontal disease, of which the level of serum immunoglobin G is reported to 8.8% correlate with PC (Ahn et al., 2012).

### Microbiota-Mediated Immunoregulation

Immune cells along with substantial microbes maintain the symbiosis between the human body and microorganisms (Mowat and Agace, 2014). Compositions of the microbiota are influenced by the immune system. Conversely, GM also plays an indispensable role in the maturation and continued education of the host immune system (Fulde and Hornef, 2014) and modulates the homeostatic state when existing an imbalance between the immune system and tumorigenesis (Kau et al., 2011; Schwabe and Jobin, 2013).

**Figure 1:**
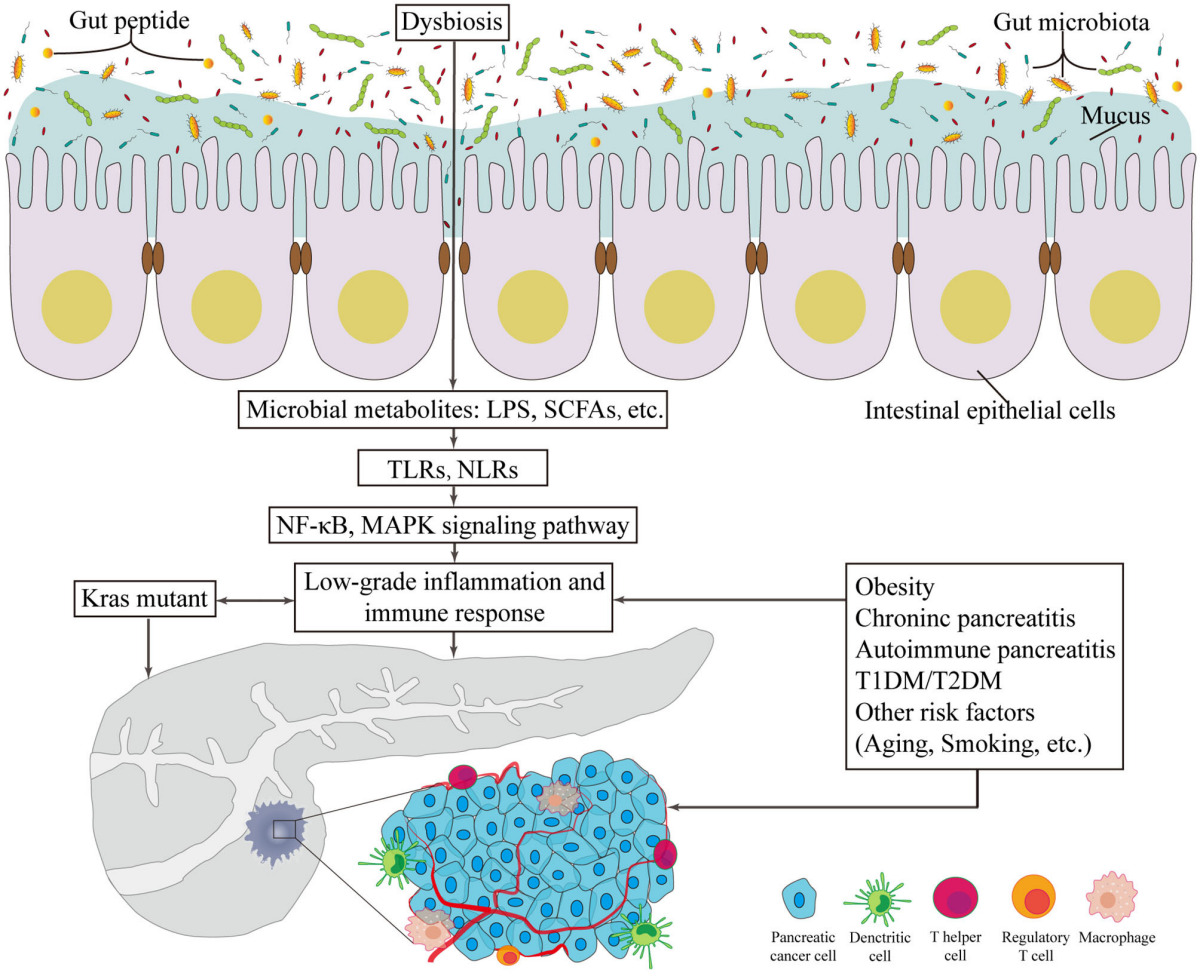
The potentially carcinogenetic roles of gut microbiota in pancreatic cancer. Disorders of gut microbiota contribute to multiple changes associated with pancreatic cancer. Locally, it lowers the thickness of intestinal mucus and the level of gut peptide. Infiltrated bacterial metabolites, such as LPS and SCFAs, can render low-grade inflammation and immune responses via TLR and NLR that activates NF-κB and MAPK signaling pathways. Besides, risk factors of pancreatic cancer provide novel consequential directions to explore the relationship between gut microbiota and pancreatic cancer. LPS, lipopolysaccharides; SCFA, short chain fatty acid; TLR, Toll-like receptor; NLR, Nod-like receptor; NF-κB, nuclear factor kappa B; MAPK, mitogen activated protein kinase; T1DM, type 1 diabetes mellitus; T2DM, type 2 diabetes mellitus.

A limited number of germline-encoded pattern recognition receptors (PRRs) exists in the innate immune system; these receptors recognize pathogens of microorganisms, known as pathogen-associated molecular patterns. TLR family plays a remarkable role in inflammation transmission and tumorigenesis acceleration once activated in response to microbiota-derived products (Zambirinis et al., 2015), such as bacterial LPS, LTA, lipoproteins, lipopeptides, flagellin, single- or doubled-stranded DNA, and CpG DNA (Kawai and Akira, 2010). In addition to detecting the conserved microbe associated molecular patterns, TLRs can also be activated by inflammation or damage associated molecular patterns (DAMPs). As downstream targets of these patterns, the NF-κB and MAPK signaling pathways are activated, which ultimately initiates cytokine production and further recruitment of proinflammatory entities and even contributes to the occurrence and development of cancer (Rakoff-Nahoum and Medzhitov, 2009) (Figure 1). Importantly, one example of protection against pancreatic carcinogenesis is to blockade the activation of TLR7 to inhibit its interactions with the STAT3, Notch, NF-κB and MAPK pathways. This effect has been successfully tested in systemic lupus erythematosus (Ochi et al., 2012). Similarly, more studies focus on another PRR, the nucleotide-binding oligomerization domain (Nod)-like receptors (NLRs). These receptors recognize microbial signals to activate caspase-1 inflammasomes and release of interleukin (IL)-1β and IL-18 (such as NLRP1, NLRP3, NLRP4) (Martinon et al., 2009). Additionally, NLRs participate in bacterial clearance by inducing the activation of NF-κB, P38 MAPK and interferon signaling; regulate autophagy-associated protein expressions; and promote autophagosome formation (such as NOD1 and NOD2) (Moreira and Zamboni, 2012; Mukherjee et al., 2018). As a result, the intestinal microbiota plays a critical role in the maturation and continued education of the host immune system (Fulde and Hornef, 2014), provides protection against pathogen overgrowth (Kamada et al., 2013), and influences host-cell proliferation (Ijssennagger et al., 2015) and vascularization (Reinhardt et al., 2012).

## GM and Risk Factors for PC

Since the initiating oncogenic factor, mutant Kras, alone cannot sufficiently explain the oncogenesis and the development of PC, additional environmental and metabolic stressors are likely required (Eibl and Rozengurt, 2019). The effects of bacterial etiology on PC could well be underestimated because the direct link between cancers and bacterial infections is not always detectable. Nevertheless, mounting evidence on the association between GM and the relative risks of PC has been presented in numerous studies (Figure 1).

### Obesity

It is no longer a novel discovery that obesity significantly increases the risk and lowers the overall survival rate of PC (Li et al., 2009). Compared to leaner counterparts, obese mice and humans present a decrease in the overall diversity of GM, which has recently been considered the main reason for alterations in metabolic signaling pathways (Mishra et al., 2016). Therein, increases in *Firmicutes* species and reductions in *Bacteroides* species are mostly obvious. Dysbiosis is associated with obesity and increased risk of cancer due to the generation of procarcinogenic microbial metabolites, low-grade inflammation and the loss of energy (Rogers et al., 2014). The modulation of gut peptides on obesity and tumorigenesis has been reported in many cancers, but multiple promising areas warrant further investigations in PC. The increasing circulating levels of inflammatory factors derived from adipose tissues in individuals with obesity and diabetes, such as IL-1, IL-6, TNF-α and monocyte chemoattractant protein-1, activate the innate immune system *via* abnormal regulation of the NF-kB signaling pathway (Creely et al., 2007), which has been strongly demonstrated to be activated in PC.

### Chronic Pancreatitis

Chronic pancreatitis is another strong risk factor (Balkwill and Mantovani, 2001), presenting as a localized or systemic inflammation. Non-alcoholic fatty pancreatic disease was recently identified as a novel clinical obesity-related disease that increases pancreatic fatty degeneration and may progress to chronic inflammation and PC (Tomita et al., 2014). Concomitantly, overgrowth of bacteria in the small intestine was commonly observed among chronic pancreatitis patients, with up to 92% of patients presenting this increase (Kumar et al., 2014; Capurso et al., 2016). Maekawa and coworkers have presented direct proof of *E. faecalis* in chronic pancreatitis (Maekawa et al., 2018). Although the association of subclinical chronic pancreatitis and PC remains ambiguous, evidence showed *E. faecalis* or LTA might aggravate chronic pancreatitis and damage pancreatic tissue by stimulating inflammatory cytokines and become potential biomarkers for PC.

### Diabetes

T2DM is associated with moderate and profound intestinal dysbiosis. Data from a metagenome analysis show an increase in certain *Lactobacillus* species but a decrease in the numbers of butyrate-producing *Roseburia intestinalis* and *Faecalibacterium prausnitzii* in stool samples from T2DM patients (Qin et al., 2012). Currently, the t2definitive causal relationships among GM, SCFAs and T2DM have been reported. One study found that the improved insulin response after oral glucose-tolerance test was related to the increase in butyrate driven by host-genetics, while the increasing risk of T2DM was causally associated with production or absorption abnormalities of propionate (Sanna et al., 2019). Moreover, studies of the environmental determinants of diabetes suggested the protective effect of SCFAs against T1DM (Vatanen et al., 2018). Mechanistically, SCFA recognition by GPR-41or−43 subsequently stimulate the secretion of gut peptides involved in food intake or glucose metabolism. Nevertheless, further extensive exploration of influences of other microbial metabolism or gut microbiomes on diabetes is warranted.

## Therapy

The use of bacterial toxins from *Streptococcus erysipelas* and Serratia species for sarcoma treatment in 1891 (Coley, 1910) established the application of bacteria or their metabolites in cancer therapy. Fecal microbiota transplantation has become a novel therapeutic approach for severe or recurrent *Clostridium difficile* infection (Kelly, 2013; Hvas et al., 2019), although more studies to identify the efficacy and safety of this approach in cancer treatments are needed. Applications of GM in cancer therapy have become increasingly prevalent.

### Chemotherapy

Although chemotherapy is efficient in inhibiting proliferation or shrinking the volume of cancer, there still exist problems in severe side effects and chemoresistance (Chabner and Roberts, 2005). Nevertheless, chemotherapy still maintains a central status in cancer therapy, especially synergized with GM. For example, oxaliplatin, a platinum preparation especially for PC chemotherapy, would be reduced without the assistance of the innate immune response activated by GM (Iida et al., 2013). Additionally, gemcitabine, the first-line chemotherapeutic drug for PC, was also shown to be affected by pyrimidine nucleoside phosphorylase and cytidine deaminase enzymes, most of which are produced by Gamma-proteobacteria and mycoplasma identified inside PDAC tumors; these data provide an individualized and targeted strategy for combating chemoresistance based on microbiota modulation (Vande Voorde et al., 2014).

Current tools designed to modify GM are quite blunt and almost all of them are focused on the toxicity of chemotherapeutic drugs rather than their efficacy. James and colleagues proposed the TIMER (translocation, immunomodulation, metabolism, enzymatic degradation and reduced diversity and ecological variation) method to summarize interactions between GM and host in order to modulate chemotherapy efficacy and toxicity (Alexander et al., 2017). Interestingly, GM is responsible for promoting the development of chemotherapy-induced mechanical hyperalgesia partly mediated by TLR4-expressing macrophages, while that pain was reduced in germ-free or antibiotics-pretreated mice (Shen et al., 2017).

### Immunotherapy

In the late nineteenth century, William Coley proposed the first immunotherapeutic concept based on pyogenic bacteria (Coley, 1991). Although immune checkpoint inhibitors have recently become fairly prominent, several negative feedback pathways due to resistance necessitates a greater focus on increasing the efficacy of immunotherapy. Currently, GM has been recognized as a possible modulator influencing immune responses in different individuals (Robert et al., 2011; Borghaei et al., 2015). Immune checkpoint inhibitors targeting programmed cell death protein 1 (PD-1) and its ligand (PD-L1) are more efficacious in cancers with Kras mutations, which still lacks targeted therapies (Ansell et al., 2015; Routy et al., 2018). A study showed that oral administration of *Bifidobacteria* combined with an anti-PD-L1 immunomodulator could induce the production of tumor-specific T cells and increase CD8(+) T cell numbers. Correspondingly, *Bifidobacterium* facilitates the effects of anti-PD-L1 via T-cell priming and peritumoral accumulation in melanoma (Sivan et al., 2015). As vigorously as the field of immunotherapy is growing, there is still no remarkable improvement in the survival of PC patients. This gap in care reflects the urgent demand for novel treatment strategies, such as considering the auxiliary effect of GM.

## Perspectives

PC is highly malignant and lacks effective treatments. GM has been recognized to involve in the inflammatory state and immune responses in PC and its associated risk factors. Targeting GM has been explored as a novel opportunity for cancer chemotherapy and immunotherapy via alterations of not only GM compounds but also molecules related to the immune response and chronic inflammation. Nevertheless, the mechanism by which certain molecules or subtypes of immune cells are targeted for microbiota-associated initiation, progression, development and treatment of PC remain unclear, leaving extensive room for further explorations.

## Author Contributions

YL and LJ contributed to the conception and design of the review. QL wrote the main text of the manuscript. MJ prepared the figures and tables. QL and MJ contribute equally to this review. All authors reviewed the manuscript.

## Conflict of Interest

The authors declare that the research was conducted in the absence of any commercial or financial relationships that could be construed as a potential conflict of interest.

## Abbreviations

CTLA-4: cytotoxic T-lymphocyte associated protein 4
DCA: deoxycholic acid
*E. faecalis*: *Enterococcus faecalis*
GM: gut microbiota
*H. pylori*: *Helicobacter pylori*
IL: interleukin
LPS: lipopolysaccharides
LTA: lipoteichoic acid
MAPK: mitogen activated protein kinase
NF-κB: nuclear factor kappa B
NLR: Nod-like receptor
NOD: nucleotide-binding oligomerization domain
PC: pancreatic cancer
PDAC: pancreatic ductal adenocarcinoma
PD-1: programmed cell death protein 1
PRR: pattern recognition receptor
SCFA: short chain fatty acid
T1DM: type 1 diabetes mellitus
T2DM: type 2 diabetes mellitus
TLR: Toll-like receptor.
